# The relationship among leadership, innovation and knowledge sharing: A guidance for analysis

**DOI:** 10.1016/j.dib.2018.04.138

**Published:** 2018-05-05

**Authors:** Hamzah Elrehail

**Affiliations:** a American University in the Emirates, Business Management (COBA), Dubai, UAE; b Girne American University, Business Management Department, Cyprus

## Abstract

This data article describes the contingent role of knowledge sharing between two leadership styles and innovation in higher education institutions in non-western countries. The dataset investigates the second-ordered (i.e. higher construct) among the relationships of study constructs, the nested relationship which gives birth to new constructs. Further, this dataset provides guidance for readers willing to conduct research in the management field. The dataset has been collected using a self-administered questionnaire obtained from the academic staff in the selected universities. The structural equation modeling (SEM) was applied using IBM SPSS AMOS. In this data article, several analysis techniques have been used. The results obtained from this dataset shows a significant relationship between leadership and innovation, and the results show that the moderation effect of knowledge sharing is partially supported.

## Specifications Table

**Table:** 

Subject area	*Business, Management*
More specific subject area	*Leadership, Innovation, Knowledge Management*
Type of data	*Table, figure*
How data was acquired	*Survey*
Data format	Analyzed, Descriptive and Statistical data
Experimental factors	– *Sample consisted of academic staff in four universities* – *As mentioned in the previous studies, the academic staff is the source of innovation* – *The contingent role of knowledge sharing is not well explored in the literature*
Experimental features	*Leadership is a well-known predictor of innovation and knowledge sharing that improves innovation in any sector*
Data source location	*Jordan*
Data accessibility	*Data is included in this article*

## Value of the data

• This dataset describes the role of leadership in enhancing innovation in the higher education sector by introducing the norms of knowledge sharing in this nested model.
• The results obtained from the dataset showed a positive relationship between leadership styles and innovation in the private universities in Jordan.
• The assumption of the effect of knowledge sharing is partially supported in this study.
• This data article provides guidance for Ph.D. and Master students for analyzing their studies in the field of management.

## 1 Data

Regarding data analyses, this data article utilized IBM SPSS AMOS, version 21. First, frequency analysis was used to generate the profiles of the subjects based on the following demographic variables: age, gender, marital status, education level, and monthly income. Following [1] recommendations, the author diagnosed for non-response errors and the missing data effect. This was achieved by comparing the demographic features of the sample relative to the general population [1]. The comparison of the current research population's demographics with those of the general population showed no significant difference. The central purpose of this dataset is to test a research model that diagnoses the simultaneous impact of Authentic leadership and Transformational leadership on process and product innovation, as well as the moderating role of knowledge sharing. Generally, speaking the aforementioned constructs are high-order or second-order constructs. A first-order construct has observed variables (i.e., the items in its measure) as indicators of the construct. The “association between indicators and their construct in a first-order construct typically assumes the indicators are observable instances and a diagram of the construct, and its indicators would show the construct specified or connected to the indicators with arrows from the construct to the indicators (a reflexive relationship)” as noted by [2], [3].

On the other hand, second-order or higher constructs have other unobserved constructs as their indicators. That is, nested constructs that give birth to new constructs, are often known as second-order constructs. In this data article, authentic leadership was manifested by the following (i.e., self-awareness, internalized moral perspective, balanced processing, relational transparency) constructs. Transformational leadership was manifested by the following (i.e., inspirational motivation, idealized influence, intellectual stimulation, individualized consideration) constructs. Knowledge sharing was manifested by the following (i.e., knowledge collecting and knowledge donating) constructs. Innovation was manifested by the following (i.e., product innovation and process innovation) constructs. Before data collection started, the researcher adopted ethical codes following Diener and Crandall's procedures [4] such as “Harm to Participants”, “Consent”, “Deception” and “Privacy”, all of these terms informed the participants in this survey.

## 2 Experimental design, materials and methods

The respondents who took part in this study were academic staff who work in universities located in the north side of Jordan. All responses were collected from four universities there. The number of valid responses was 173. The questionnaire was designed to probe the respondents’ demographic characteristics, including their gender, marital status, age, experience, academic qualifications, academic position, and their university name. To confirm the expected second-order factor of authentic leadership, transformational leadership, knowledge sharing, and process and product innovation, the author conducted a confirmatory factor analysis (CFA) on the data following Hinkin's [5] recommendations. In doing this, the author utilized maximum likelihood estimation to analyze the multivariate normality. First, a series of CFA were conducted for each construct to assess the first-order construct model fit; that is, the relationship between scale items and their construct in a first-order construct was evaluated. Subsequently, CFA for the whole measurement model was conducted to assess whether second order constructs assumes and "drives" the indicator from the first-order construct.

Several model fit indices were assessed, namely: *χ*2 measure, the Goodness of Fit Index (GFI), the Normed Fit Index (NFI), Incremental Fit Index (IFI), Comparative Fit Index (CFI), and the Root Mean Square Residual (RMR). In Table 1, the goodness of fit for the four second order constructs yielded accepted results: CMIN (*χ*2) values all had significant *p* values, *p* < .001, GFI values were closer to 1, and 1 = perfect fit, NFI values were closer to 1, and 1 = perfect fit, IFI values were closer to 1, and 1 = perfect fit, CFI values were closer to 1, and 1 = perfect fit, RMR values were less than .060, and values < .060 represent perfect fit, relative (*R*.*χ*2) were between 1 and 5 which were the accepted benchmark used in prior research [6], [7], [8], [9], [10], [11], [12]. Next, The Normed Fit Index (NFI), Incremental Fit Index (IFI), Comparative Fit Index (CFI), and Root Mean Square Error of Approximation (RMSEA) were chosen as they can predict the “absolute measure of fit based on the non-centrality parameter” better than RMR, especially in instances where we have second-order constructs. In Table 2, the goodness of fit for the four second-order constructs yielded a good fit for the dataset: CMIN (*χ*2) had a significant *p* value, *p* < .001, NFI value was closer to 1, and 1 = perfect fit, IFI value was closer to 1, and 1 = perfect fit, CFI value was closer to 1, and 1 = perfect fit, RMSEA values were less than or equal to .080 represent perfect fit, relative (*R*.*χ*2) value between 1 and 5 is acceptable. See Tables 1 and 2.

**Table 1:** First order CFA and goodness fit indices.

Construct	Sub-constructs	GFI	NFI	IFI	CFI	RMR	CMIN	d*f*	*R*.*χ*2
**Transformational Leadership**	Inspirational Motivation	.85	.87	.92	.92	.049	251.33	113	2.22
	Idealized influence								
	Intellectual Stimulation								
	Individualized consideration								
**Authentic Leadership**	Self-Awareness	.87	.88	.92	.92	.052	174.95	59	2.97
	Balanced processing								
	Relational Transparency								
	Internalized Moral Perspective								
**Knowledge Sharing**	Knowledge Collecting	.98	.97	.99	.99	.022	15.73	13	1.21
	Knowledge Donating								
**Innovation**	Product Innovation	.92	.93	.96	.96	.051	90.13	42	2.15
	Process Innovation								

Note: *R*.*χ*2 = CMIN/d*f*.

**Table 2:** Second-order CFA and goodness fit indices.

Constructs pairs	NFI	IFI	CFI	RMSEA	CMIN_(df)_	*R*.*χ*2
One factor test	.51	.60	.60	.115	3546.33_(1080)_	3.28
						
Authentic Leadership	.70	.81	.81	.080	2201.17_(1054)_	2.09
Transformational Leadership						
Knowledge Sharing						
Innovation						

Note: *R*.*χ*2 = CMIN/d*f*.

In this data article, a two-step approach was utilized. One, all measures for the measurement model were subjected to CFA to understand the structure and underlying interrelationships of the complex [13]. In addition, this would provide assess construct, convergent and discriminant validity. Furthermore, composite reliability and internal consistency for the measurement model would be assessed following [14], [15] recommendation. Across the study, measurement models, factor and item loadings all exceeded .50, ranging from .50 to .84 with *t*-values greater than 11.13. The average variance extracted by each latent variable was above .48, suggesting evidence of convergent validity among our measures. Discriminant validity was checked using [15] criterion. AVE values were supposed to be above .50, but transformational leadership and knowledge sharing AVEs were less than .50, [15] stated that this is not a problem as the CR values were all above .70.

The constructs composite reliabilities (CR) ranged from .86 to .94, all above the threshold of .60. The reliability of each variable was assessed through a reliability analysis with the aid of Cronbach's alpha. Reliability “pertains to the consistency of a measure and is inversely related to the degree to which a measure is contaminated by random error.” Cronbach's (*α*) were above the cutoff point of .70 [16]. In addition to evidence of convergent and discriminant validity, the results showed that the measures were reliable. See Table 3 below.

**Table 3:** Measurement model convergent and discriminant validity.

Variables	*α*	CR	AVE
Authentic Leadership	.93	.93	.51
Transformational Leadership	.93	.94	.48
Knowledge Sharing	.86	.86	.47
Innovation	.92	.93	.56

Following [17] recommendations, intra-class correlations analysis (ICC), with the aid of two-way mixed and absolute agreement definitions, were used to assess the level of agreement between-groups. Intra-class coefficients (ICCs) were estimated based on an unconditional random coefficient model in order to estimate the relative amount of between-person and within-person variation. That is, whether groups can be differentiated on the variables under investigation. Single and average measures were observed. For Authentic leadership (ICC = .51 and .93; *p* = .00); Transformational leadership (ICC = .47 and .94; *p* = .00); Knowledge sharing (ICC = .47 and .86; *p* = .00); and Product & process innovation (ICC = .54 and .93; *p* = .00). These results indicate that it was appropriate to analyze the data because it appears that the effects observed in the present study are attributable to employee's perceptions and not necessarily a particular university. This approach has been employed by a number of studies published in top journals [18], [19].

Multicollinearity is a linear relationship between two or more independent variables, diagnosing the potential effect of multicollinearity in a given dataset can be achieved through variance inflation factor (VIF). VIF quantifies how much the variance is inflated in the coefficient estimate. According to [20] “VIF values of 5 or 10 and above indicates a multicollinearity problem”. The results obtained from the dataset depicts that the VIF values of the current research variables were below the aforementioned benchmark. Generally speaking, the findings suggest that multicollinearity is not a major concern with the dataset, and that we can continue with our analyses. In the next stage, we’ll present the results of correlation path analysis and moderation effect among the study constructs. See Tables 4 and 5.

**Table 4:** Means, standard deviations (SD), and correlations of study variables.

Variables	Mean	SD	1	2	3	4
Authentic Leadership	3.59	.76	–			
Transformational Leadership	3.70	.72	.822**	–		
Knowledge Sharing	3.92	.66	.590**	.564**	–	
Product & Process Innovation	3.34	.83	.533**	.521**	.472**	–

Note: SD, standard deviation.

** Correlations are significant at the .01 level.

**Table 5:** Path analysis and moderation effect.

Independent variable	Dependent variable	*β*	S.E	*t*-value	*p*
Transformational Leadership	Product & Process Innovation	.274	.072	3.797	^***^
Authentic Leadership	Product & Process Innovation	.203	.068	2.988	.003
Knowledge Sharing	Product & Process Innovation	.287	.079	3.640	^***^
***Interaction term***					
Transformational Leadership	Product & Process Innovation	.373	.069	5.391	^***^
(Transformational Leadership * Knowledge sharing)	Product & Process Innovation	.313	.032	9.768	^***^
Authentic Leadership	Product & Process Innovation	.152	.065	2.223	.020
(Authentic Leadership * Knowledge sharing)	Product & Process Innovation	− .242	.035	− 6.848	^***^

Notes: ^**^ Significant at the *p* < 0.01 level (two-tailed); ^***^ Significant at the *p* < 0.001 level (two-tailed).

## 3 Academic and managerial implication of this data article

Our data article has a number of academic and managerial implications. First, top management should increase the number of training programs inside the universities, especially for authentic leadership and leadership terminology in general. Second, top management should provide an atmosphere that increases the contextual effect of knowledge sharing and “trust” as mentioned by [3], [21]. For the academic part, this data article provides clear guidance for Ph.D. and Master students in the field of management how they can analyze their papers, dissertation and thesis. This style of analysis is highly recommended to follow, particularly if the researchers are willing to publish a paper in reputable journals.
