# Supplementary material for: The relationship among leadership, innovation and knowledge sharing: A guidance for analysis
Source: Data Brief. 2018 May 5;19:128–33. doi: 10.1016/j.dib.2018.04.138 (PMC5992979; doi:10.1016/j.dib.2018.04.138)
Supplement: Supplementary file 1 — Supplementary material [file mmc1.docx]

The authors declare that No conflict of interest related to this data article
